# Supplementary material for: Deterministic transition of enterotypes shapes the infant gut microbiome at an early age
Source: Genome Biol. 2021 Aug 24;22:243. doi: 10.1186/s13059-021-02463-3 (PMC8383385; doi:10.1186/s13059-021-02463-3)
Supplement: Supplementary file 1 — Additional file 1: Tables S1-S3, Figures S1-S13. Supplementary tables and figures. [file 13059_2021_2463_MOESM1_ESM.pdf]

Table S1. Characteristics of infants included in this study.

Nation	Samples	Subjects	Status (term/preterm)	Gestation (weeks)	Sex (male/female)	Delivery (vaginal/c-section)	Breastfeed (yes/no)	Data type
Australia	818	442	818/0	-	-	-	-	16S
Bangladesh	1288	41	1292/0	-	-	-	-	16S
Brazil	124	7	124/0	-	-	-	-	16S
China	500	285	290/210	31±1.3	99/111	128/258	210/0	16S/WGS
Estonia	483	78	483/0	-	210/273	450/33	394/89	16S
Finland	2246	139	2246/0	-	1277/969	2091/155	1657/582	16S
India	331	14	331/0	-	-	-	-	16S
Ireland	680	197	545/135	38±3.5	395/285	289/365	404/276	16S
Italy	229	46	94/135	-	-	-	94/0	16S/WGS
Japan	485	72	301/184	37±3	298/172	282/188	-	16S
Luxembourg	85	18	85/0	38.1±1.6	57/28	37/48	65/20	WGS
Netherlands	915	120	915/0	39.5±1.1	422/493	556/359	745/170	16S
Norway	2683	12	2683/0	-	-	-	-	16S
Peru	488	22	488/0	-	-	-	-	16S
Russia	623	72	623/0	-	378/245	545/78	478/79	16S
South Africa	148	7	148/0	-	-	-	-	16S
USA	1650	384	447/1203	29.5±4.6	355/277	104/525	271/605	16S/WGS
**Sum**	**13776**	**1956**	**11909/1867**	**36±5**	**3491/2853**	**4482/1869**	**4318/1821**	

Table S2. The number of samples assigned to the four enterotypes.

	**Enterotype 1 (Firmicutes)**	**Enterotype 2 (*Bifidobacterium*)**	**Enterotype 3 (*Bacteroides*)**	**Enterotype 4 (*Prevotella*)**	**Sum**
Year 1	3062	4036	3046	0	10144
Year 2	0	791	1403	329	2523
Year 3	0	437	603	69	1109
**Sum**	**3062**	**5264**	**5052**	**398**	**13776**

Table S3. Statistical tests of the transition of enterotypes associated with clinical factors.

	Year 1	Year 2	Year 3
Number of subjects shifted	549	133	21
Number of subjects fixed	382	169	82
Sex (P value, chisq.test)	0.7484	0.9099	0.4595
Gestation age (P value, wilcoxon.test)	0.4725	0.3529	-
Birth mode (P value, chisq.test)	0.277	0.2734	-
Breastfeeding status (P value, chisq.test)	0.07634	0.4884	0.4926

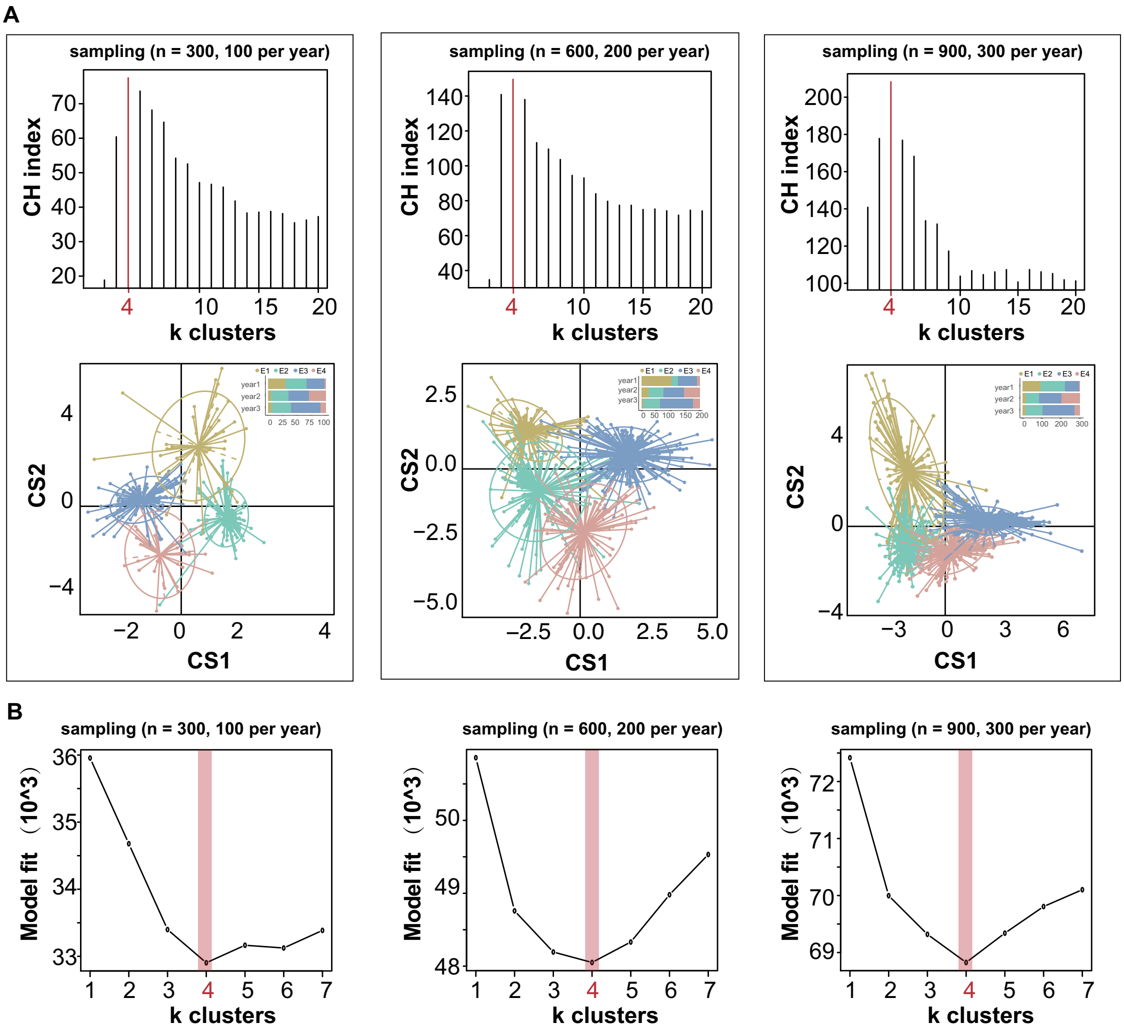

Fig S1. Enterotype clustering based on random sampling. (A) Random sampling from 3 years using partitioning around medoid (PAM) protocol. The number of samples from each year is 100 (left), 200 (middle), 300 (right), respectively. On the top row, the X axis indicates optimal number of clusters which was calculated using the Calinski–Harabasz index. The bar-plot shows the number of enterotypes in each year. (B) Enterotype clustering using Dirichlet multinomial mixtures (DMM) approach. The X axis indicates optimal number of clusters which was determined based on the lowest Laplace approximation score. The red bar highlights the optimal number of clusters in each subset.

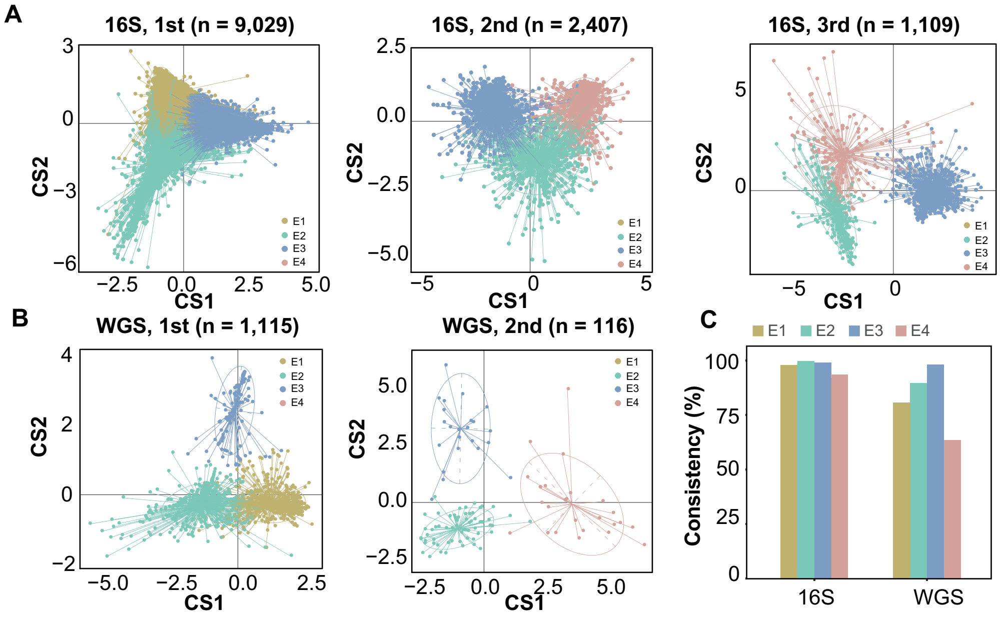

Fig S2. Enterotype clustering based on 16S rRNA and metagenomic data, respectively. (A) Enterotype clustering based on 16S rRNA gene sequencing data. (B) Enterotype clustering based on metagenomic data. (C) The consistency between the enterotypes clustered by 16S (left four bars) or metagenomic (right four bars) data and the enterotypes clustered by all data.

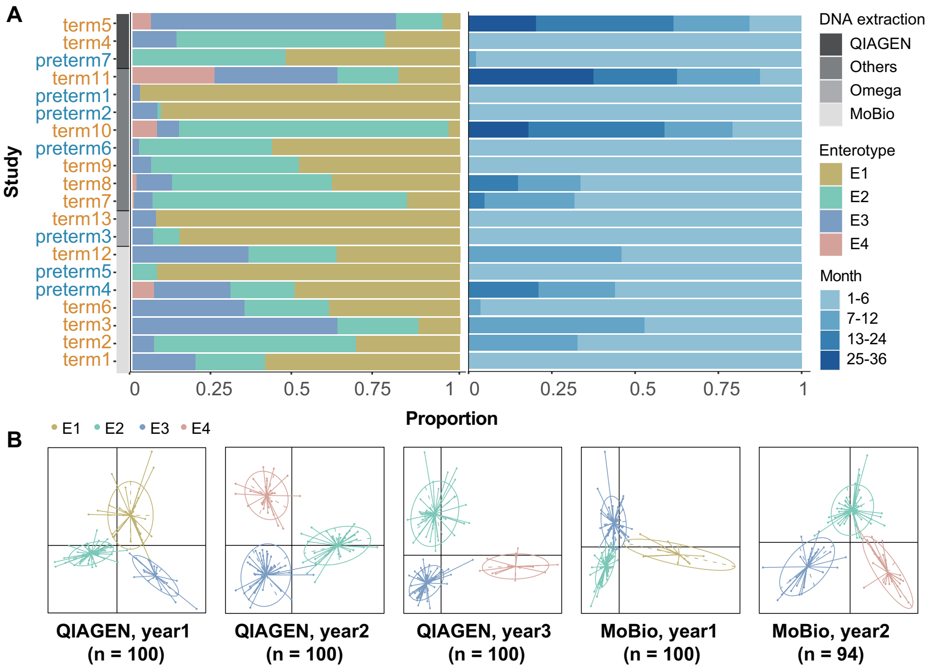

Fig S3. The effect of "study" on the enterotype clustering analysis. (A) The composition of enterotypes (left) and the composition of developmental stages (right) in each study. The grey bar on the left of the panel represents different products of DNA isolation method. (B) Enterotype clustering on fecal samples processed by different DNA isolation kits (QIAGEN, MoBio). Note: there are no 3rd year samples in the MoBio group.

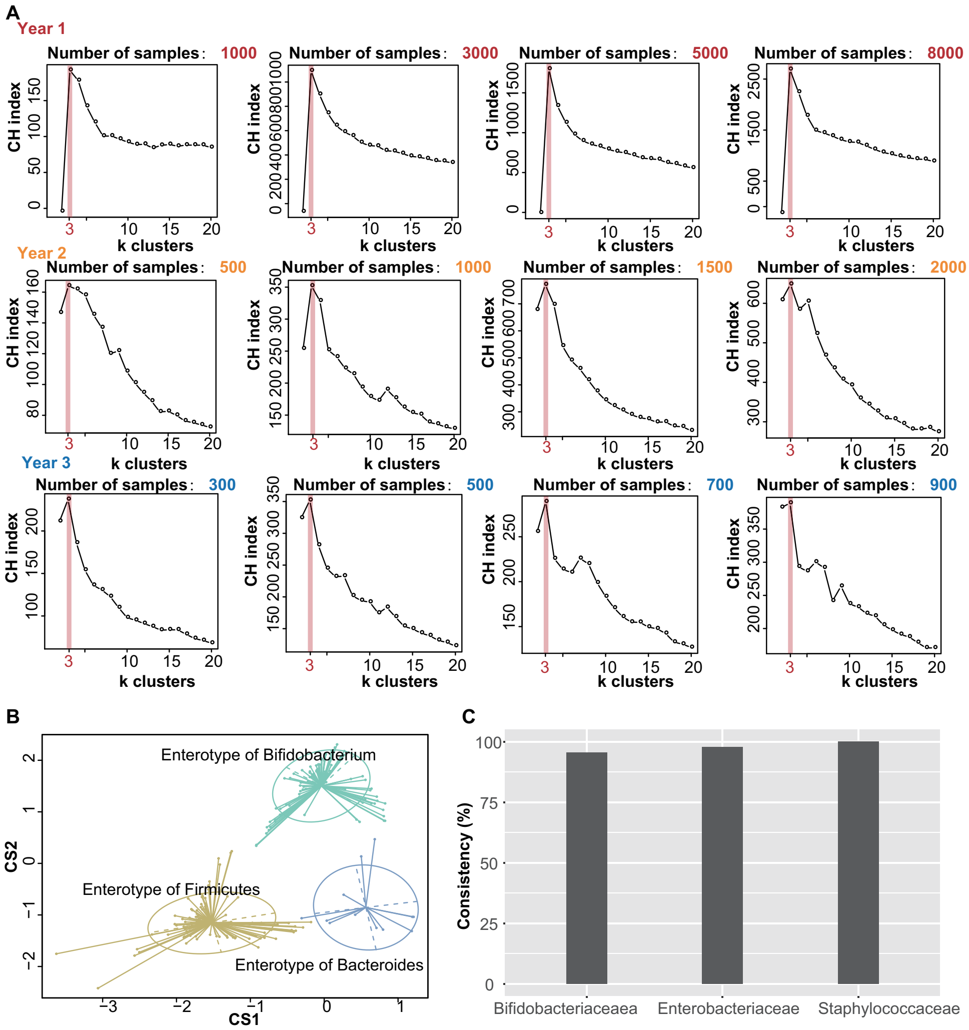

Fig S4. Enterotype clustering with different sample sizes and different cohorts. (A) Different samples were selected randomly for verifying the stability of enterotype clustering. The X axis indicates optimal number of clusters which was calculated using the Calinski–Harabasz index. The red bar highlighted in each plot indicates the optimal number of clusters in each subset. (B) Enterotypes formed in our study using the Takahiro's datasets. (C) The overlaps of enterotype clusters between our study and the previous study[1].

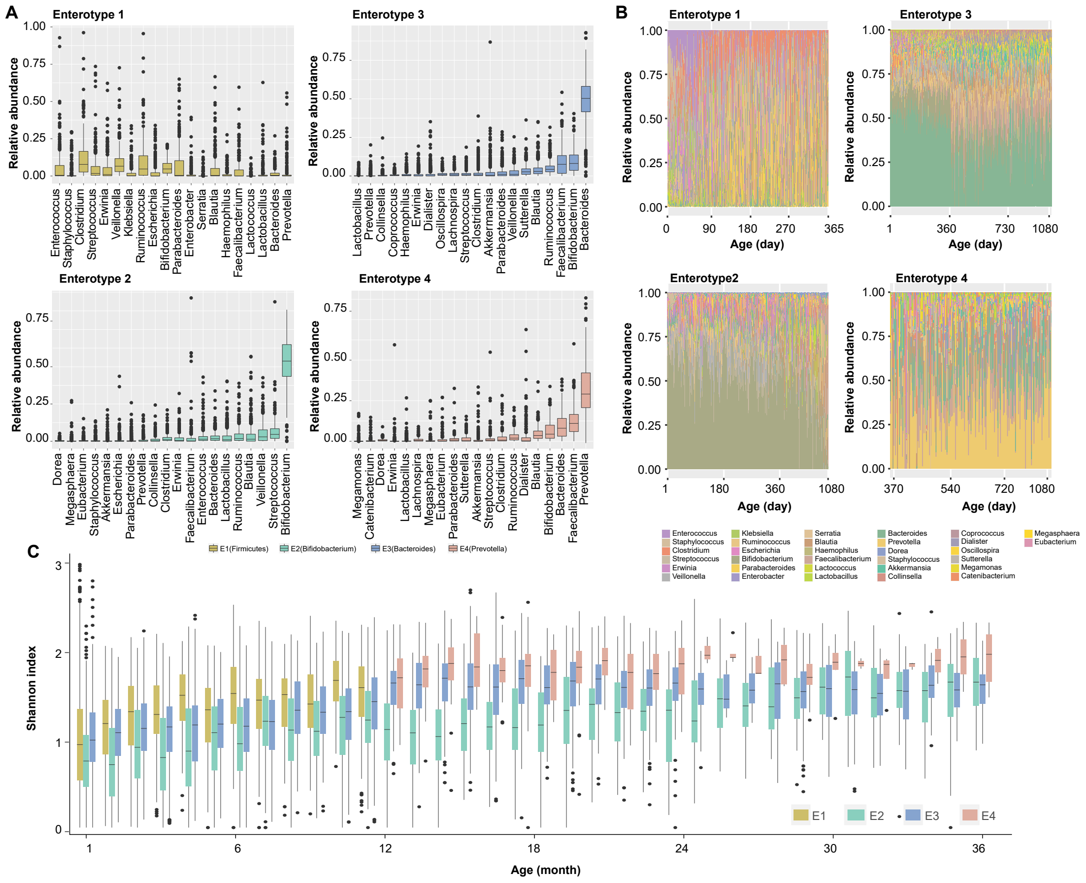

Fig S5. Community characteristics of four enterotypes associated with chronological ages. (A) Relative abundance of top 20 genus. (B) Relative abundance of dominant taxa at the genus level. (C) Alpha diversity based on Shannon index in different age groups.

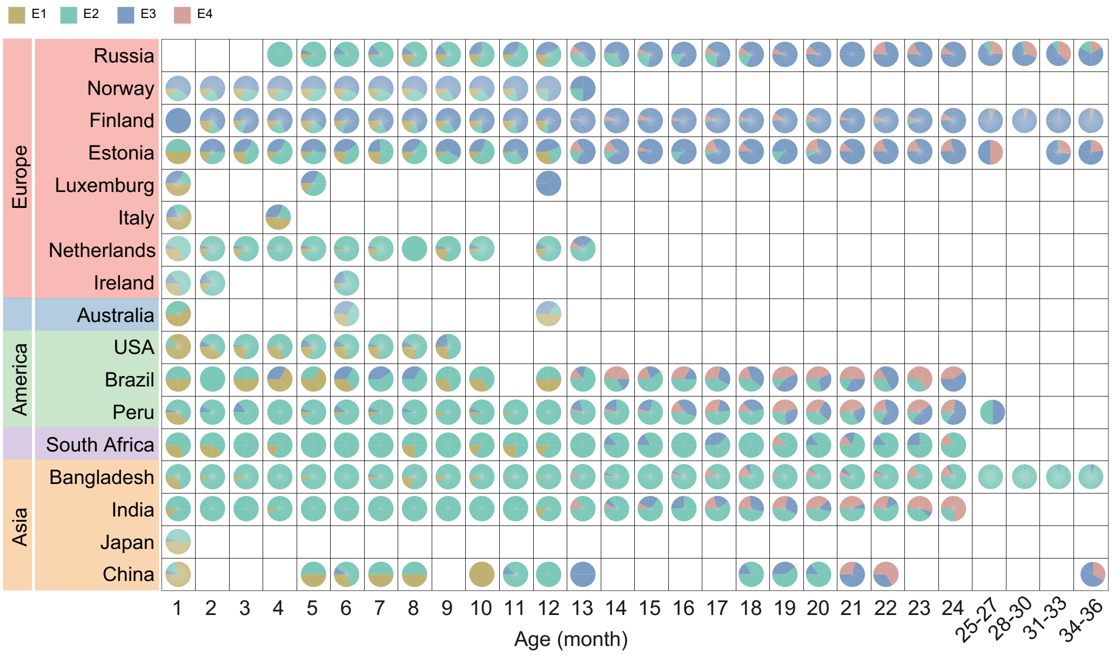

Fig S6. Geography-related pattern of enterotypes based on full-term datasets (n = 11,909).

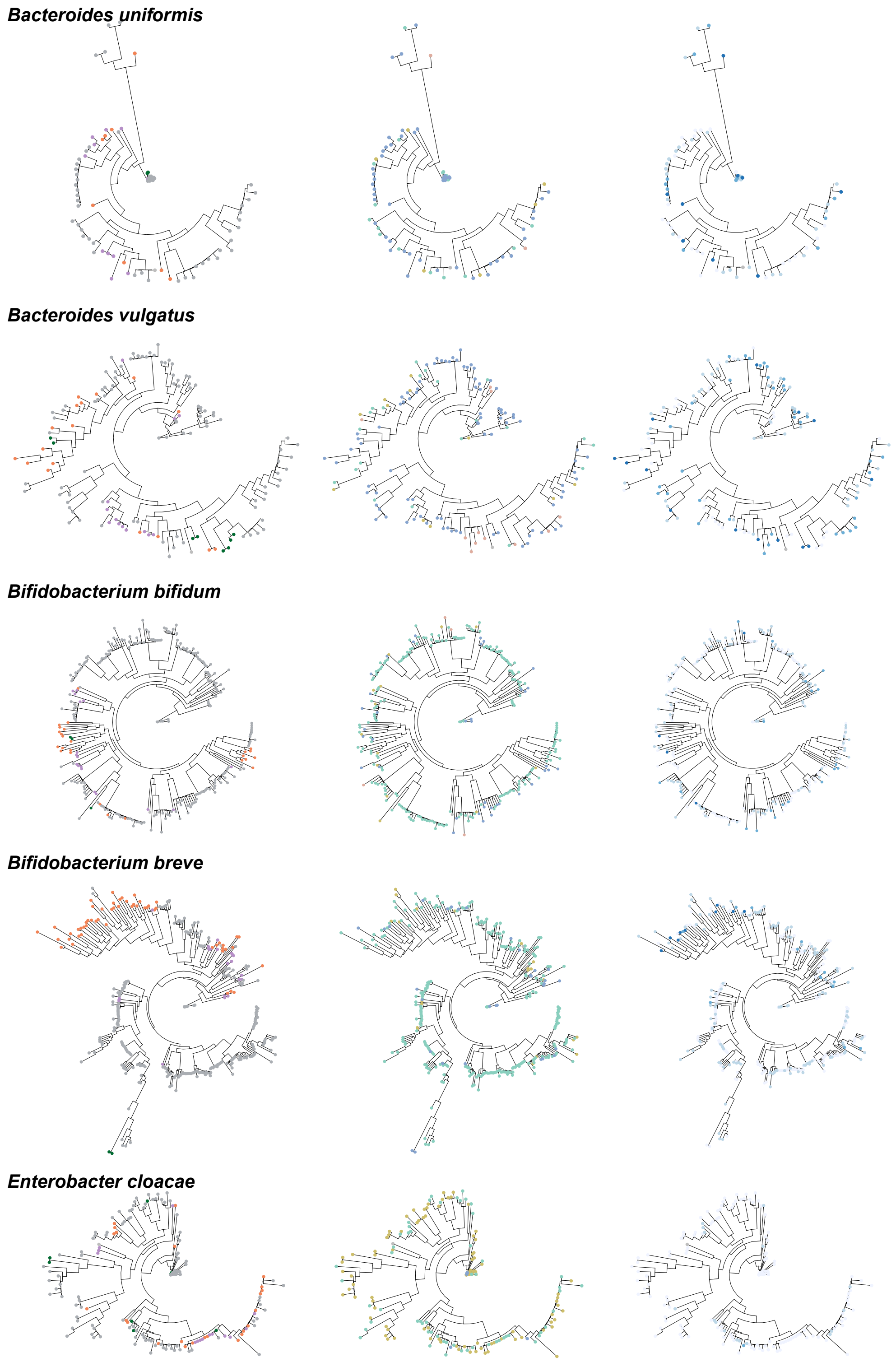

Fig S7. Phylogenetic trees of different species denoted with country (left), enterotype (middle) and developmental stage (right). Each node in the phylogenetic tree indicates a specific strain come from each infant. The SNV-based analysis and tree construction was implemented using StrainPhlAn.

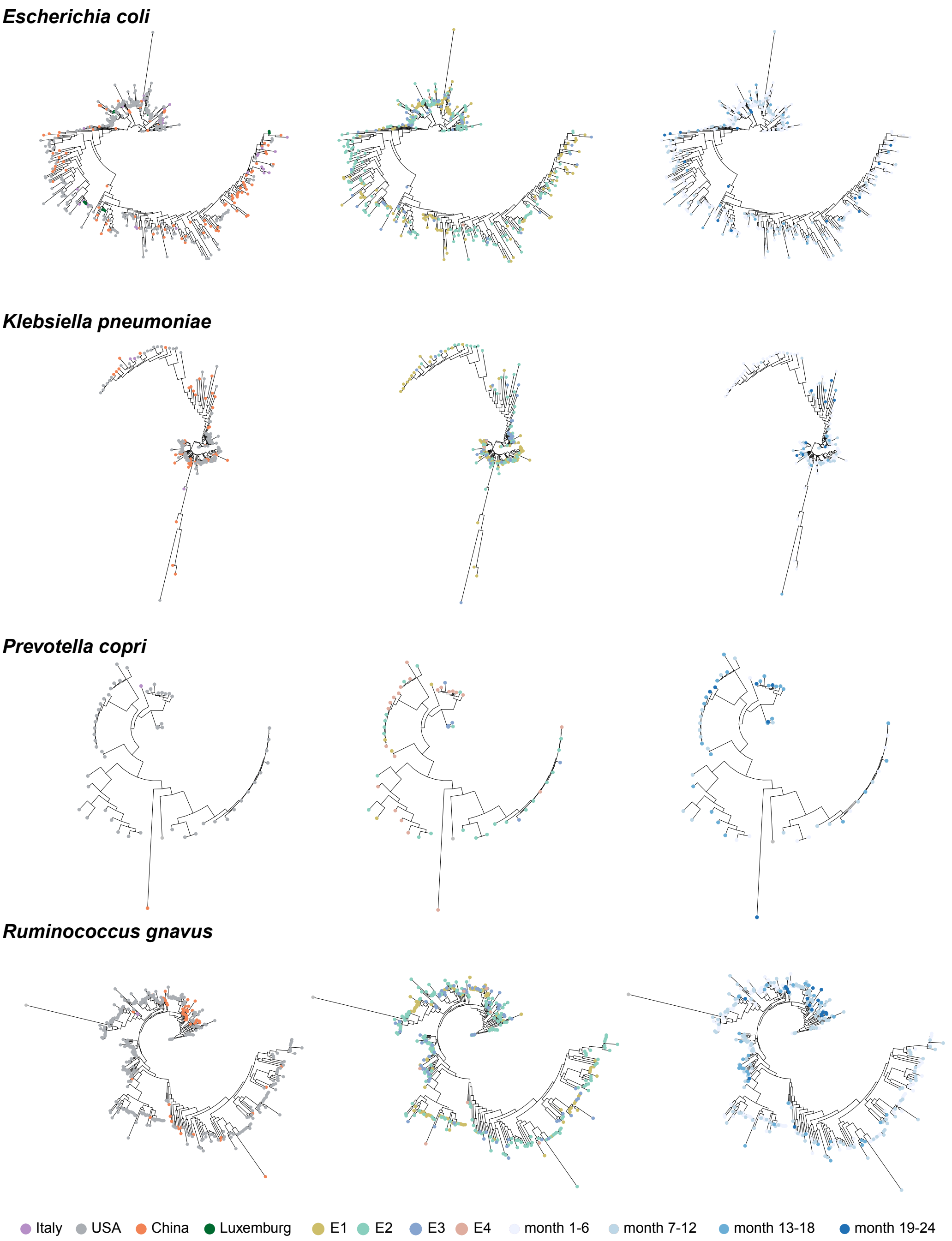

Fig S7-continued.

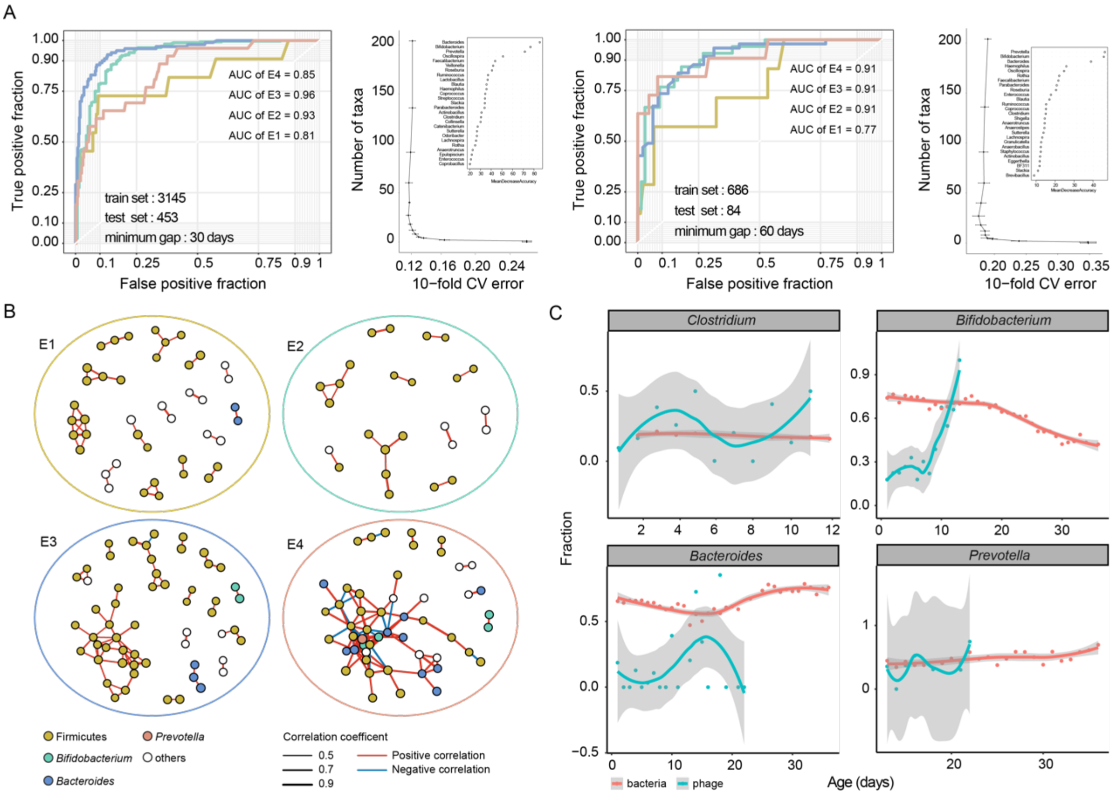

Fig S8. Influencing factors and outcomes associated with enterotype transition. (A) Prediction of enterotype transition based on random forest model. The minimum gap is 30 and 60 days, respectively. Results of 10-fold Cross Validation are presented on the right. (B) Co-occurrence network of four enterotypes. Each node indicates a specific species and each edge indicates the interaction between two species. The size of edges scale with the correlation coefficient. Only correlation coefficients greater than 0.5 (for enterotype 4, greater than 0.8) presented. (C) The correlation between four enterotype taxa and their associated bacteriophages.

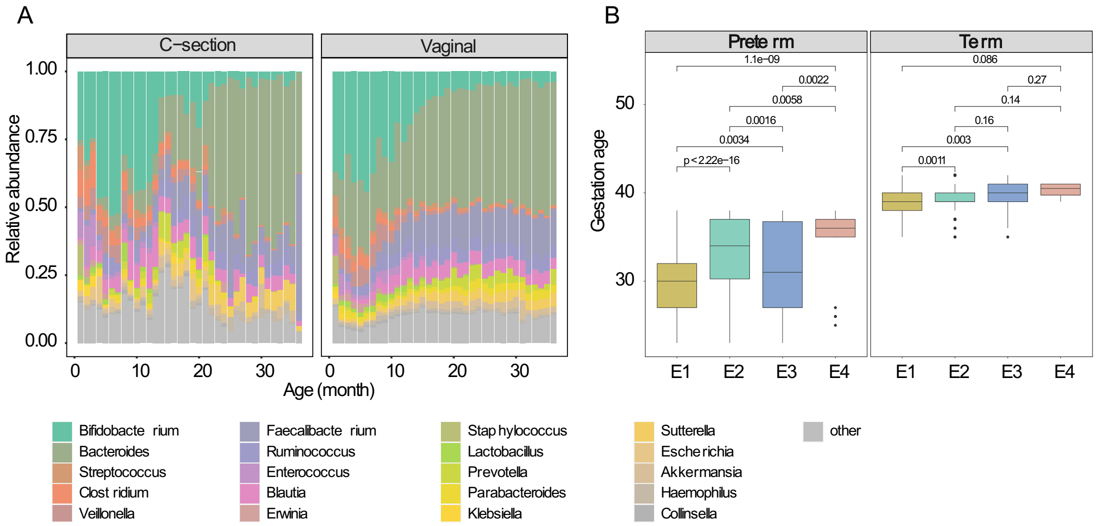

Fig S9. Variation of gut microbiota and enterotypes associated with clinical factors. (A) The relative abundance of the top 20 most abundant genera in all subjects over 36 months in different birth modes, i.e. C-section (left) or vaginal delivery (right). (B) The distribution of infants with different gestation age, i.e. preterm (left) or full-term (right).

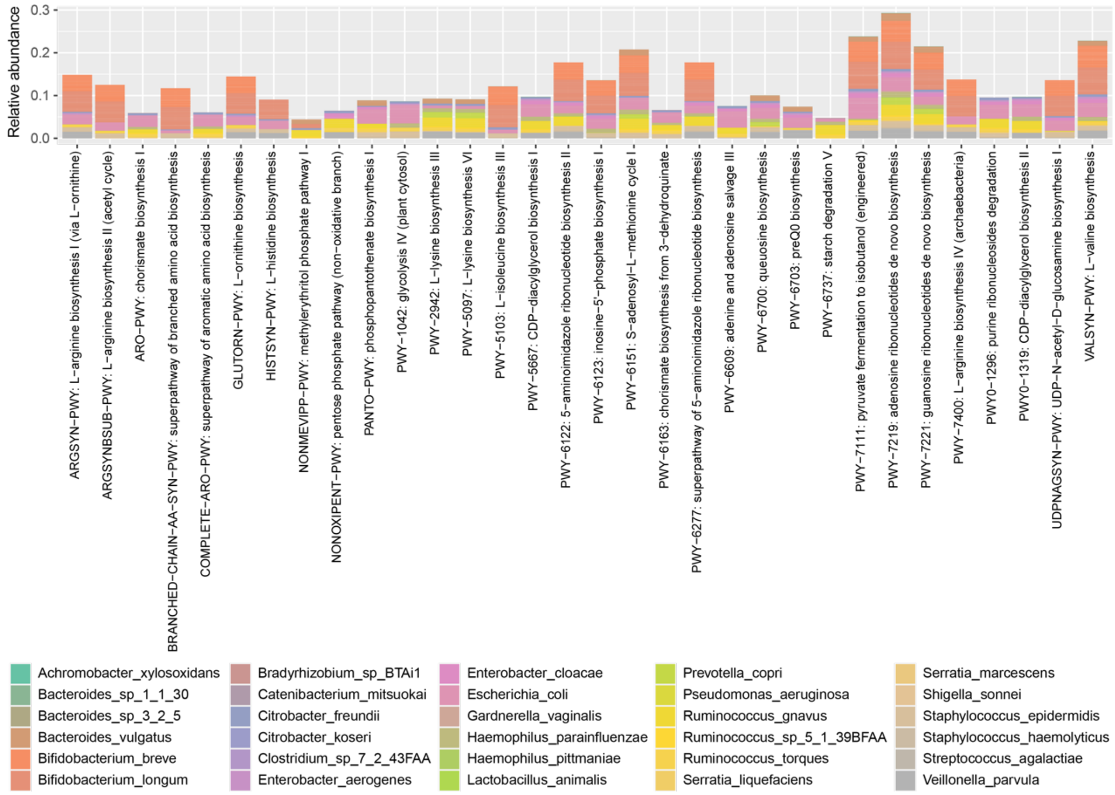

Fig S10. The top 30 contributing bacterial species to each pathway. Contributions of the top 30 species in 32 most significant pathways between enterotypes. Contributions were calculated with HUMAnN2.

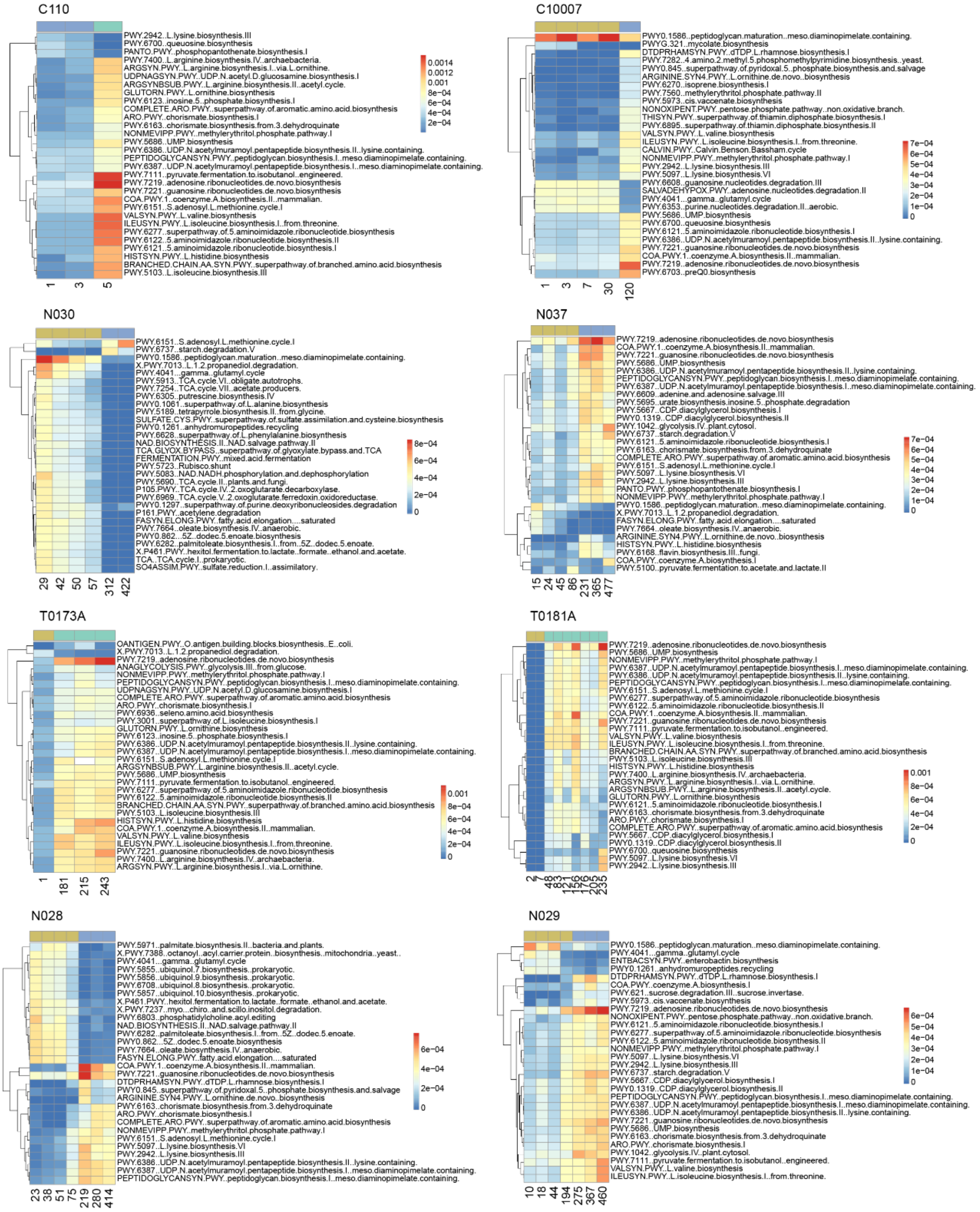

Fig S11. The associations between functional changes and transition of enterotypes in individual subjects. Heatmaps indicate the variation of the 30 most significantly different pathways of enterotypes in the first two years. Bar plots indicate the transition of enterotypes over time.

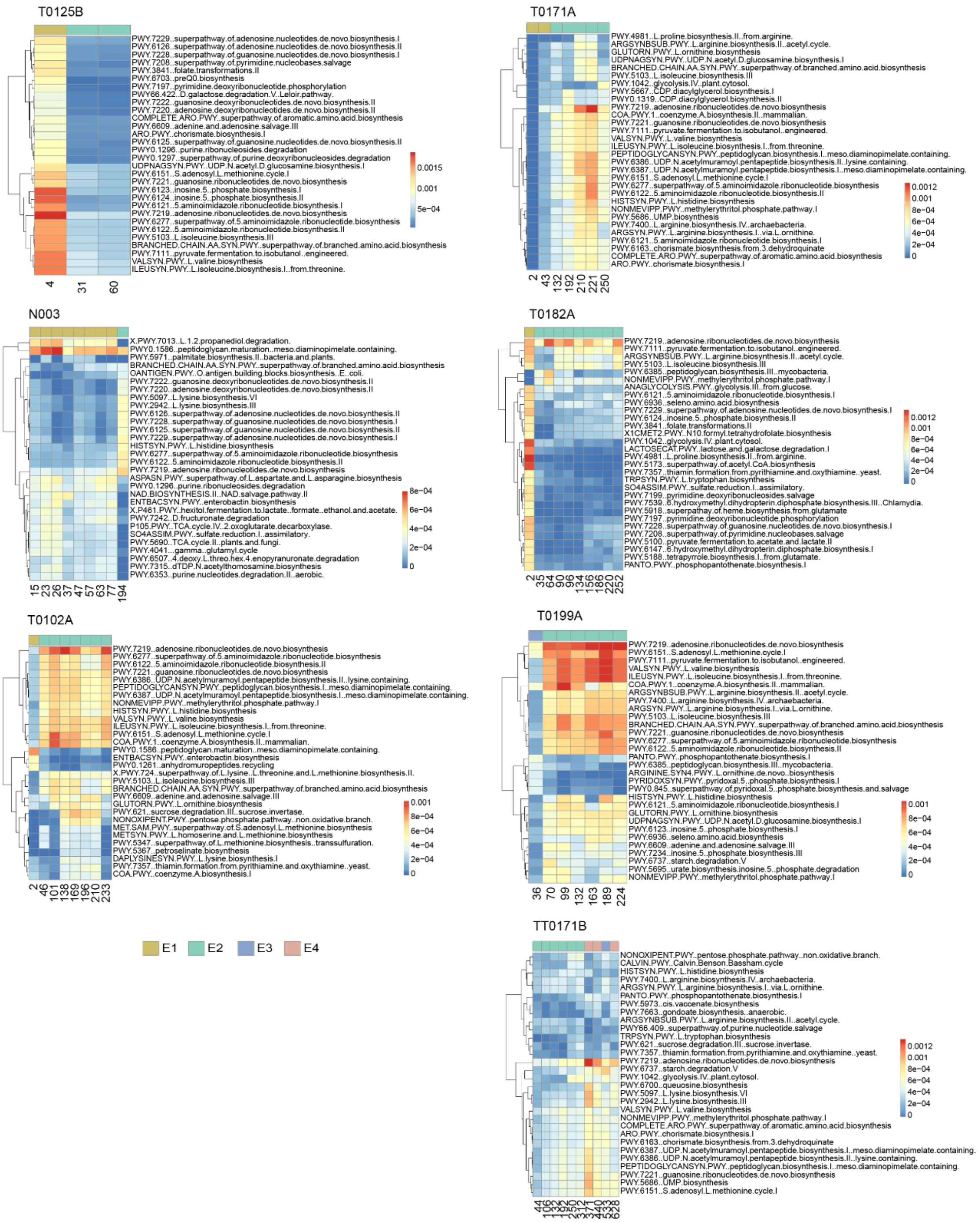

Fig S11-continued.

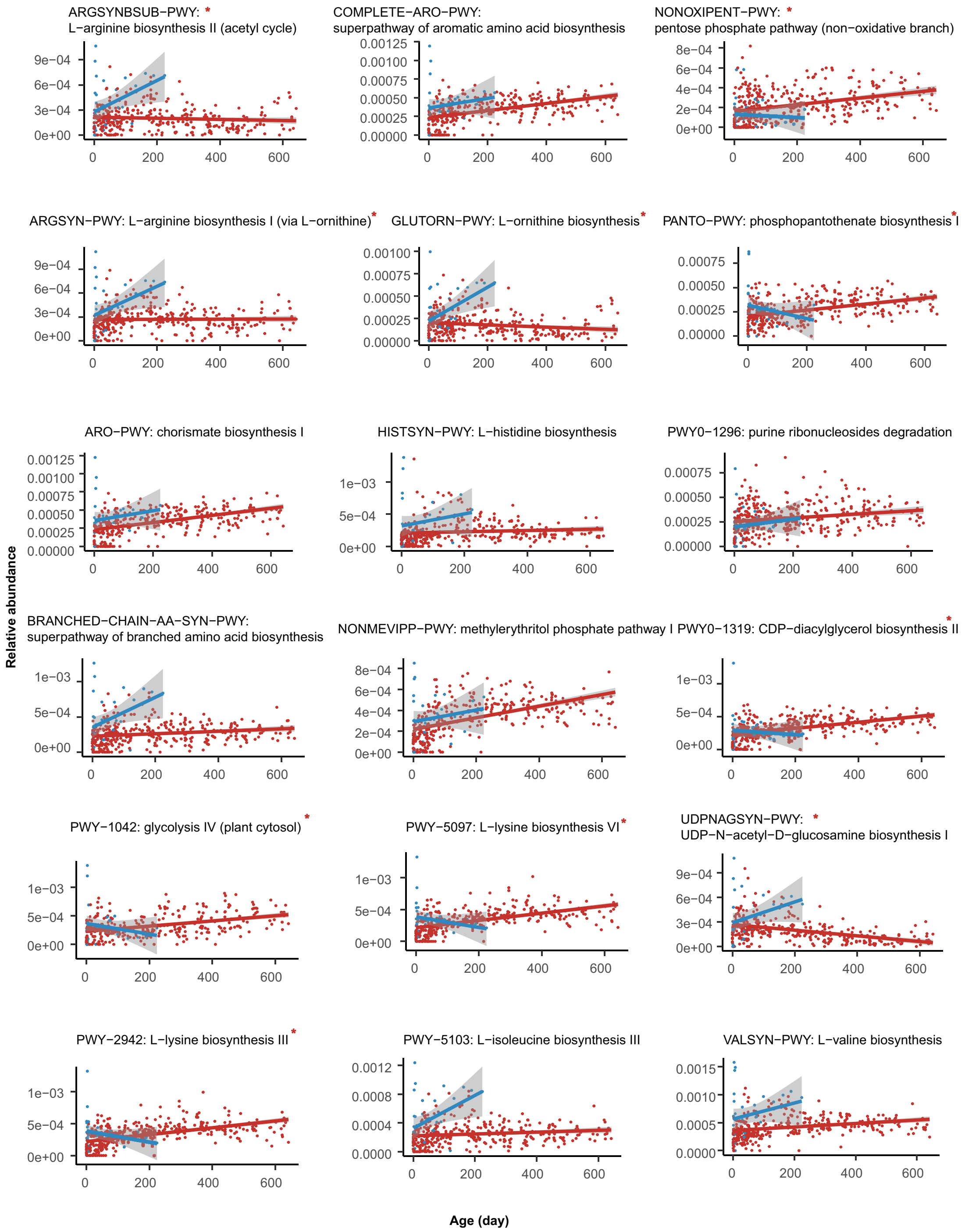

Fig S12. Variations of function features over time in infant's gut microbiota. Red lines indicate infant groups which started with E1 or E2 and transited to E3 or E4 and blue lines indicate infant group started with E3 or E4 and transited to E1 or E2. The X axis indicates the chronological ages in days. The Y axis indicates the relative abundance of each pathway. The red asterisks in plots indicate opposite trend between two subgroups.

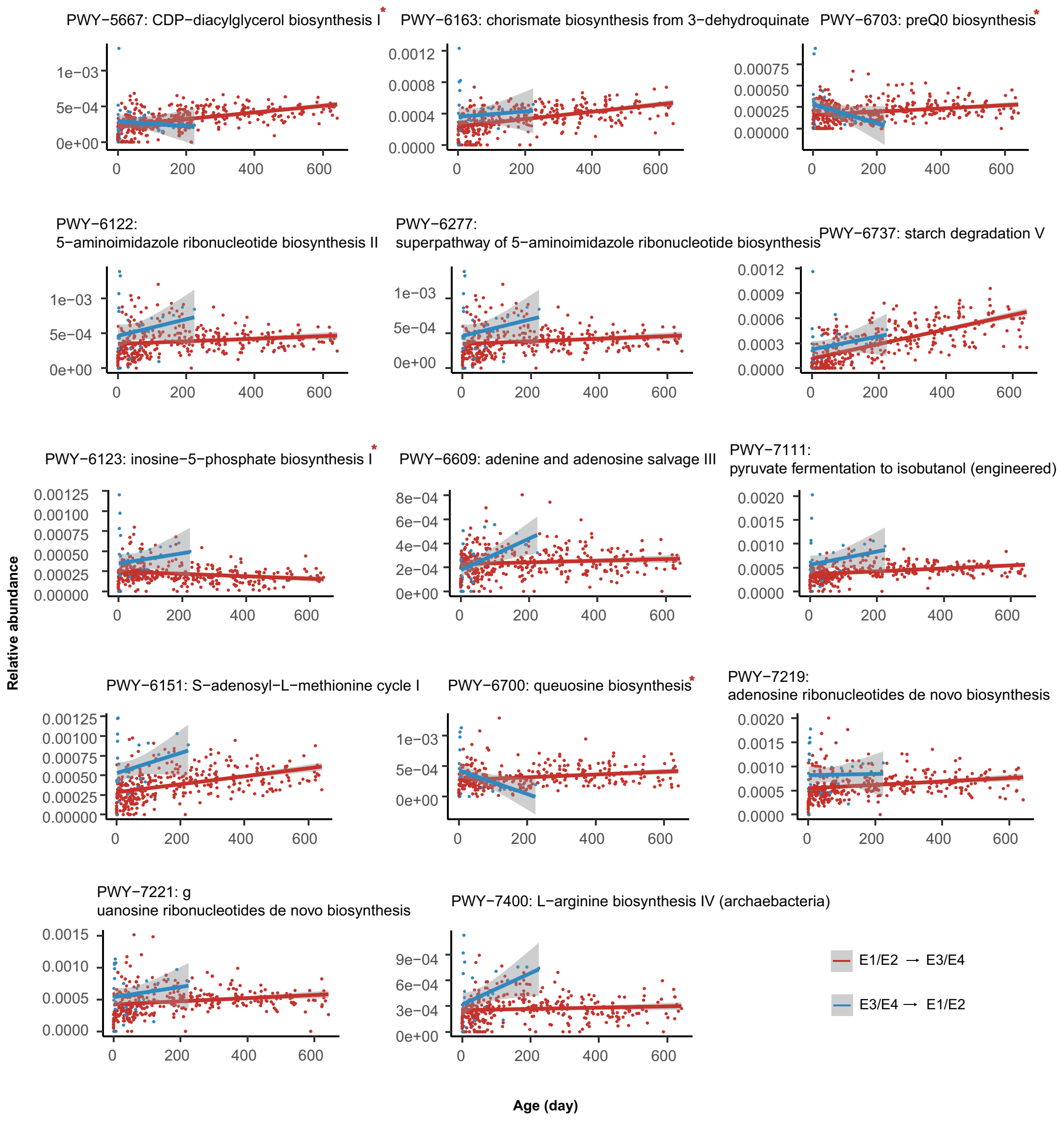

Fig S12-continued.

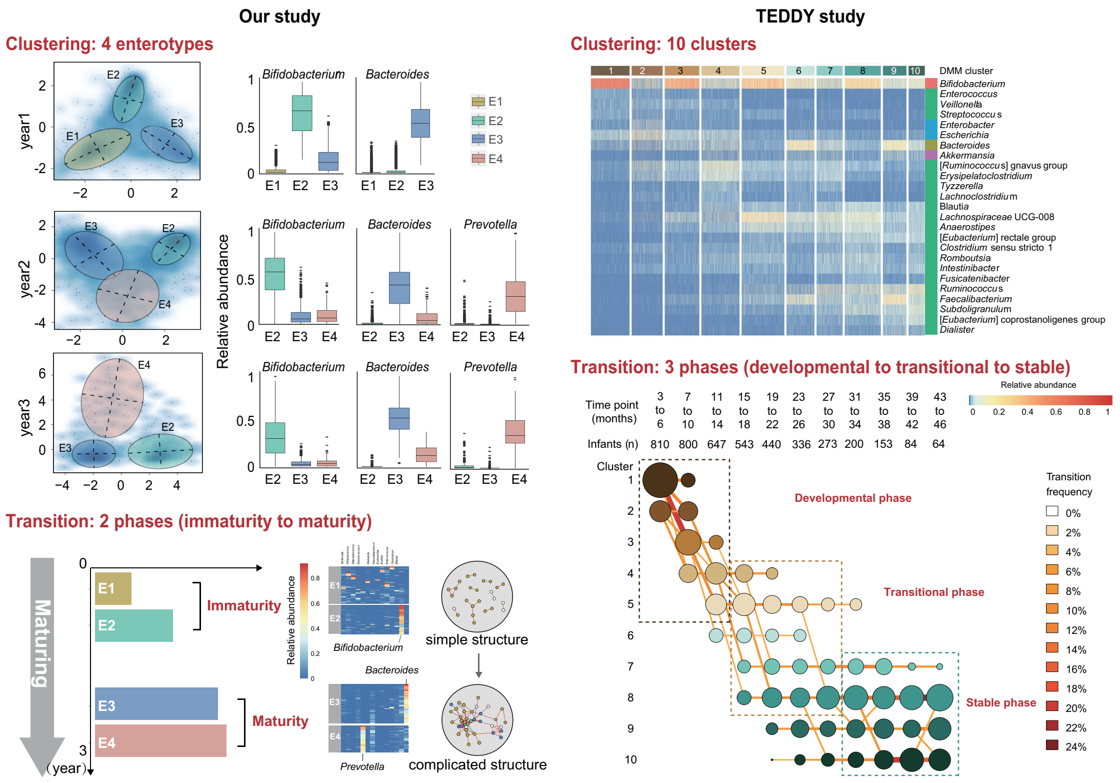

Fig S13. The differences between our study (left) and TEDDY study (right). Subfigures in the right panel are derived from the TEDDY study.
